# FAS-Dependent Cell Death in α-Synuclein Transgenic Oligodendrocyte Models of Multiple System Atrophy

**DOI:** 10.1371/journal.pone.0055243

**Published:** 2013-01-25

**Authors:** Christine L. Kragh, Gwenaëlle Fillon, Amanda Gysbers, Hanne D. Hansen, Manuela Neumann, Christiane Richter-Landsberg, Christian Haass, Bernard Zalc, Catherine Lubetzki, Wei-Ping Gai, Glenda M. Halliday, Philipp J. Kahle, Poul H. Jensen

**Affiliations:** 1 Department of Biomedicine, Aarhus University, Aarhus, Denmark; 2 Laboratory for Alzheimer's and Parkinson's Disease Research, Department of Biochemistry, Ludwig Maximilians University, Munich, Germany; 3 Neuroscience Research Australia and University of New South Wales, Sydney, New South Wales, Australia; 4 Laboratory of Functional Neurogenetics, Department of Neurodegeneration, Hertie Institute for Clinical Brain Research, University of Tübingen, Tübingen, Germany; 5 Institute for Neuropathology, University of Zürich, Zürich, Switzerland; 6 Department of Biology, University of Oldenburg, Oldenburg, Germany; 7 Centre de Recherche de l'Institut du Cerveau et de la Moelle épinière, Université Pierre et Marie Curie, Paris, France; 8 Department of Human Physiology and Centre for Neuroscience, Flinders University School of Medicine, Bedford Park, South Australia, Australia; 9 German Center for Neurodegenerative Diseases, Tübingen, Germany; University of Maryland School of Pharmacy, United States of America

## Abstract

Multiple system atrophy is a parkinsonian neurodegenerative disorder. It is cytopathologically characterized by accumulation of the protein p25α in cell bodies of oligodendrocytes followed by accumulation of aggregated α-synuclein in so-called glial cytoplasmic inclusions. p25α is a stimulator of α-synuclein aggregation, and coexpression of α-synuclein and p25α in the oligodendroglial OLN-t40-AS cell line causes α-synuclein aggregate-dependent toxicity. In this study, we investigated whether the FAS system is involved in α-synuclein aggregate dependent degeneration in oligodendrocytes and may play a role in multiple system atrophy. Using rat oligodendroglial OLN-t40-AS cells we demonstrate that the cytotoxicity caused by coexpressing α-synuclein and p25α relies on stimulation of the death domain receptor FAS and caspase-8 activation. Using primary oligodendrocytes derived from PLP-α-synuclein transgenic mice we demonstrate that they exist in a sensitized state expressing pro-apoptotic FAS receptor, which makes them sensitive to FAS ligand-mediated apoptosis. Immunoblot analysis shows an increase in FAS in brain extracts from multiple system atrophy cases. Immunohistochemical analysis demonstrated enhanced FAS expression in multiple system atrophy brains notably in oligodendrocytes harboring the earliest stages of glial cytoplasmic inclusion formation. Oligodendroglial FAS expression is an early hallmark of oligodendroglial pathology in multiple system atrophy that mechanistically may be coupled to α-synuclein dependent degeneration and thus represent a potential target for protective intervention.

## Introduction

Multiple system atrophy (MSA) is a sporadic and progressive neurodegenerative disease that presents with motor abnormalities like akinesia, rigidity and postural instability. No effective symptomatic treatment is currently available. Unlike the other α-synucleinopathies of Parkinson's disease (PD) and dementia with Lewy bodies (DLB), which are characterized by neuronal aggregates of α-synuclein (α-syn), MSA is neuropathologically characterized by glial cytoplasmic inclusions (GCIs) containing aggregated α-syn in oligodendrocytes [1], [2], [3]. The presence of α-syn in oligodendrocytes is enigmatic as these cells do not normally express α-syn, but its accumulation may be due to aberrant expression or transcellular uptake from neurons. Still, the pathogenic potential of α-syn in oligodendrocytes has been demonstrated in transgenic (tg) mice overexpressing human α-syn under the control of oligodendrocyte-specific promoters [CNPase, MBP and PLP] [4], [5], [6]. These tg mice develop α-syn accumulations in oligodendrocytes and exhibit oligodendroglial and neuronal pathology or increased sensitivity to toxins [7], [8]. Changes in oligodendrocyte morphology are observed in MSA prior to α-syn accumulation and aggregation. These changes comprise proteolysis of myelin basic protein (MBP) in myelin and enlargement of oligodendroglial cell bodies with accumulation of the oligodendrocyte-specific protein p25α prior to the accumulation of α-syn [9].

In addition to oligodendrocytic myelin loss and α-syn accumulation, MSA patients display considerable neuronal loss accompanied by astrogliosis and microgliosis [10], [11]. This is recapitulated in tg mouse models overexpressing human α-syn in oligodendrocytes [5], [6]. *In vivo* and *in vitro* studies of MBP-hα-syn mice and primary oligodendrocytes from CNPase-hα-syn mice point to a pathogenic role of transcellular secretory substances [12], [13]. FAS (CD95) is a plasma membrane death domain receptor that activates the extrinsic apoptotic pathway upon interaction with the FAS ligand (FASL) and plays an important role in immune-related cell removal [14]. FAS has also been implicated in degenerative processes in the central nervous system in general [15] and in oligodendrocyte cell death in an experimental model of multiple sclerosis (MS) [16].

In the present study, we demonstrate a functional role of autocrine signaling through FAS in the degenerative pathway of α-syn aggregation in the oligodendroglial OLN cell line. Primary oligodendrocytes from hα-syn tg mice [4] display α-syn-dependent sensitization to the apoptotic effect of FASL. Analysis of post mortem MSA tissue demonstrates an increased FAS expression in brain homogenates and in oligodendrocytes containing early-stage GCIs. We hypothesize that autocrine and paracrine FAS signaling may represent an active contributor to the neurodegeneration observed in MSA.

## Materials and Methods

### Plasmids and transfection

pcDNA3.1 zeo(-) plasmid expressing human p25α was produced by PCR with pET-11d vector containing the human p25α gene as template [17]. The product was inserted into pcDNA3.1 zeo(-) vector, which was transformed into competent *E. coli* DH5α cells to select positive clones for sequencing. The chosen clones were cultured and plasmid DNA was purified. The construct was confirmed by sequencing. Transient transfections were performed with Fugene-6 Transfection Reagent (Roche, Mannheim, Germany) according to the manufacturer's protocol.

### Oligodendrocyte cell line experimentation

OLN-t40-AS rat oligodendroglial cells are based on the OLN-93 cell line derived from primary Wistar rat brain glial cultures [18]. Cells were kept at 37°C under 5% CO_2_ and grown in DMEM (Lonza, Verviers, Belgium) supplemented with 10% foetal calf serum (FCS), 50 U/ml of penicillin and 50 µg/ml of streptomycin. OLNt40AS cells were maintained in 50 µg/ml geneticin. These cells express human α-syn and develop α-syn aggregate-dependent degeneration upon coexpression with the pro-aggregatory p25α protein [19].

For inhibition of caspase activity, cells were treated with 20 µM of caspase-3, -8, and -9 inhibitors, Ac-DEVD-CHO, Ac-IETD-CHO or Ac-LEHD-CHO (Bachem, Weil am Rhein, Germany), for 1 h prior to transfection with p25α and during the post transfection period. For inhibition of FAS signaling, cells were pretreated with 1 µg/ml of mouse monoclonal anti-FAS antibody (clone ZB4, Upstate Biotechology, Temecula, USA) for 1 h prior to transfection with p25α and during the post transfection period.

α-Syn aggregate-dependent degeneration was quantified by measuring the development of microtubule (MT) retraction in OLN-t40-AS cells expressing p25α as previously described [19]. In brief, cells were processed for immunofluorescence microscopy using rabbit polyclonal anti-p25α antibody [17] and mouse monoclonal anti-α-tubulin (Sigma, Steinhelim, Germany), counterstained with DAPI and analyzed by fluorescence microscopy. MT retraction was defined as a retraction of MT from the cellular processes to the perinuclear region resulting in intense α-tubulin staining surrounding the nucleus. MT retraction was quantified by counting p25α-positive cells with a clear perinuclear localization of MT compared to the total number of p25α-positive cells. In each experiment, 200 transfected cells localized in five randomly chosen microscopic fields were examined at 100 times magnification. Two independent investigators counted the cells blind to treatment conditions. Student's *t* test was used to determine whether differences between groups were significant.

### Purification and culturing of oligodendrocytes

Transgenic oligodendrocyte progenitor cells were purified from perinatal (PLP)- α-syn, (PLP)-eGFP and WT C57Bl6 mouse brain as previously described [20]. Briefly, mixed glial cultures were obtained from two days old mouse forebrains. Mice were killed by decapitation, and forebrains were dissected before being dissociated, first mechanically and then by digestion with 0.1% trypsin (15 min at 37°C). Cells were washed in Hank's balanced salt solution, passed through 150 µm and 63 µm nylon meshes and layered on a percoll density gradient. The oligodendrocyte progenitor-enriched fraction was then centrifuged and resuspended in DMEM containing 10% FCS. Cells were plated onto poly-L-lysine coated glass coverslips or plastic 24-well plates for 20 min at 37°C in an atmosphere containing 5% CO_2_ to allow cell adhesion. 500 mL of Bottenstein and Sato medium, supplemented with 1% FCS, 1% penicillin-streptomycin and 10 ng/ml recombinant platelet-derived growth factor (PDGF)-AA (Peprotech) was added to the oligodendrocyte progenitor cultures. After 7 days in culture, the medium was changed to a differentiating medium (proliferating medium without PDGF).

To stimulate the extrinsic apoptotic pathway, oligodendrocyte cultures were treated for 18 h with 5 ng/ml soluble FAS ligand (sFASL) or membrane-bound FAS ligand (mFASL) (Upstate Biotechnology). To block signaling through the FAS receptor, cells were preincubated with neutralizing anti-FAS antibody (1 μg/ml) (clone ZB4). Cells were stained with Hoechst (Sigma) to identify apoptotic nuclei. For quantitative assessments, 500 oligodendrocytes from three randomly selected fields were analyzed. Student's *t* tests or one-way ANOVA followed by Dunnett's test were used to determine whether differences between groups were significant.

### Immunocytochemistry

For double immunostaining of surface and cytoplasmic antigens, cultures were rinsed once with phosphate-buffered saline (PBS), and fixed with 4% paraformaldehyde in PBS for 5 min at room temperature (RT). After washing with PBS, fixed cells were blocked with 10% FCS for 30 min and incubated overnight at 4°C with primary antibodies diluted in blocking solution. For double immunostaining involving only cytoplasmic antigens, cultures were rinsed once with PBS and fixed with 4% paraformaldehyde in PBS for 15 min at RT. After washing with PBS, fixed cells were blocked with 10% FCS in PBS for 30 min and incubated for 1 h with primary antibodies diluted in blocking solution containing 0.02% Triton-X-100. Cells were washed, incubated for 1 h with secondary antibodies and counterstained with Hoechst 33342. To examine the extent of non-specific binding, primary antibody was omitted. After washing in PBS, coverslips were mounted in fluoromount (Southern Biotechnology Associates).

Cells were assessed using an Eclipse TE300 fluorescence microscope (Nikon) and 20X or 40X objectives, with phase contrast microscopy performed with a 40X objective. Images were acquired with simple PCI software (Compic Inc. Imaging Systems). To demonstrate co-localization, cells were visualized using 63X or 100X oil-immersion objectives with an axioplan2 imaging Zeiss microscope or with an LSM510 Zeiss confocal microscope. Images were acquired with FluoUp Mercator software (Explora Nova) and LSM10 Meta (Zeiss), respectively.

### Human brain samples

Approval was obtained to assess frozen brain tissue from MSA cases (*n* = 13) and age-matched controls (*n* = 15), collected with consent and ethics approval through the Australian Brain Bank Network. MSA cases were autopsy confirmed and controls had no significant neuropathology and no evidence of neurological or psychiatric disease. All cases were collected within 36 hrs of death (average of 15±11 hrs). The average age of the MSA group was 68±8 years with average disease duration of 7±2 years. The average age of the control group was 76±10 years and was not different from the MSA group (*p* = 0.17).

### Immunohistochemistry

Tissue was sampled from frontal, temporal and parietal association and primary cortices, basal ganglia, basal forebrain and brainstem. Sections were deparaffinized in xylene, rehydrated through serial changes of ethanol gradients and boiled in citrated buffer containing 1 mM EDTA, pH 8.0, for 10 min. Endogenous peroxidase was blocked by incubating sections in 1% H_2_O_2_/50% methanol for 10 min. Sections were blocked with 20% normal horse serum for 1 h and incubated overnight at RT with anti-FAS (1∶40) (NovaCastra, Newcastle, UK) diluted in TBS pH 7.4 containing 1% normal horse serum. Sections were subsequently incubated with secondary antibody (biotinylated donkey-anti-mouse antibody) for 1.5 h, Vectastain ABC (Vector Labs) for 1 h and developed in DAB solution (SigmaFast) for 10 min. Sections were counterstained with haematoxylin, dehydrated and coverslipped.

Double-labeling immunofluorescence was performed on 10 μm-thick formalin-fixed paraffin-embedded sections from the putamen. The primary antibodies were mouse monoclonal anti-α-syn (mAb42, Transduction Labs, diluted 1∶200), rabbit polyclonal anti-FAS (C20, Santa Cruz Biotechnology, diluted 1∶50) and a rat polyclonal antibody raised against human p25α (produced in-house, diluted 1∶50). The secondary antibody combinations were goat anti-mouse Alexa fluor 488 (diluted 1∶500) with goat anti-rabbit Alexa fluor 568 (diluted 1∶250) for detecting FAS with either α-syn or p25α. All primary and secondary antibodies were diluted in 0.1 M Tris buffer. Sections were first deparaffinized in xylene and rehydrated in graded ethanols. Antigen retrieval was performed using 90% formic acid for 3 min (for detection of α-syn) and boiling in 0.1 M citrate buffer pH 6.0 for 3 min (for FAS and p25α detection). Sections were pretreated with 5% H_2_O_2_/50% ethanol and 10% normal horse serum in 0.1 M Tris buffer prior to primary antibody incubation at RT overnight. After 3×5 min washes in 0.1 M Tris buffer, sections were incubated in secondary antibody for 2 hrs at RT followed by 3×5 min washes with 0.1 M Tris buffer. Sections were coverslipped using Vectashield mounting medium for fluorescence (Vector Laboratories). To determine specificity of the antibodies using these methods, primary antibody was excluded from the procedure. In these experiments no labeling was produced. Quantification of FAS immunoreactivity was determined in two types of GCIs: those with large, bright, obvious p25α immunoreactivity and those with α-syn immunoreactivity. Double-labeling fluorescent images were captured using constant settings on a Nikon Microscope ECLIPSE 90i confocal microscope with a Nikon D-ECLIPSE C1 high-resolution camera to ensure adequate image processing for evaluation. 404 large, bright p25α-positive GCIs were evaluated for colocalisation with FAS. 374 α-syn-positive GCIs were evaluated for colocalization with FAS as well as for FAS immunoreactivity within but remote to the α-syn-positive GCIs in cells.

### Frozen tissue sampling and fractionation

Half of the hemisphere was freshly sectioned and frozen blocks stored at −80°C prior to fractionation. 100–200 mg of frozen brain tissue from white matter under the precentral gyrus (as this region contains GCIs but does not contribute to clinical presentation and is without substantial atrophy and degeneration) was used. Tissue was homogenized in 10x vol of TBS-homogenisation buffer [50 mM Tris, 125 mM NaCl pH 7.4, 5 mM EDTA, 0.02% NaN_3,_ 1X protease inhibitor cocktail (Roche)], sonicated 2×10 s on ice and then cleared by centrifugation at 120,000 g for 2 hrs at 4°C. The supernatant was referred to as the TBS-soluble extract. The pellet was washed twice with homogenization buffer, resuspended in 10x vol solubilisation buffer with 5% SDS, sonicated for 2×10 s and centrifuged at 100,000 g for 30 min at 23°C. The supernatant was referred to as the SDS soluble fraction. All protein concentrations were determined using the BCA method.

### Quantitative Western Blotting

Standard Western blotting methods were used for quantifying the relative amounts of FAS, p25α and α-syn proteins in SDS soluble protein fractions from human MSA and control brains. 20 μg of SDS-soluble protein was denatured at 70°C for 10 min and separated by SDS-PAGE. Proteins were then transferred onto polyvinylidene difluoride (PVDF) membranes (BioRad Laboratories). The membranes were blocked with 5% powdered skim milk in TBS-T (10 mM Tris-HCl, pH 7.5, 150 mM NaCl and 1% Tween-20) for 1 h. Membranes were probed overnight at 4°C using primary antibodies diluted in blocking buffer (monoclonal mouse anti-α-syn at 1∶4000 (BD Transduction Labs), polyclonal rabbit anti-FAS (Santa Cruz Biotechnology) at 1∶2000, polyclonal rabbit anti-p25α at 1∶12,000 [17], and monoclonal mouse anti-β-actin at 1∶50,000 (Sapphire Biosciences)). Membranes were washed three times in TBS-T buffer and probed with HRP-conjugated secondary antibodies diluted in blocking buffer for 1 h. Immunoreactivity was visualized by chemiluminescence using an ECL detection system captured onto Hyperfilm. Films were scanned and the intensity of each band was quantified using NIH Image J software (National Institutes of Health) with expression normalized to β-actin Statistical differences were analyzed using SPSS (IBM SPSS Statistics 18) and a *p* value less than 0.05 accepted as significant. Group comparisons were performed using Mann Whitney U tests and correlations between variables were performed using Spearman Rho tests.

## Results

### α-synuclein-dependent degeneration in oligodendroglial cells involves FAS signaling

We coexpressed human p25α and human α-syn in the rat oligodendrocyte OLN cell line to model the situation observed in dystrophic oligodendrocytes in MSA [9]. As already described the coexpression caused a rapid α-syn-dependent degeneration that was followed by a protracted presentation of characteristics of apoptosis where the rapid cytoskeletal changes and the ensuing apoptotic characteristics were ameliorated by a caspase-3 inhibitor [19]. Active caspase-3 is a downstream effector caspase in the apoptotic pathway that is activated by upstream caspases as part of different signaling pathways, e.g. caspase-9 as part of the mitochondrial pathway and caspase-8 by ligand binding to membrane-associated death domain receptors like FAS (reviewed in [21], [22]).

**Figure 1:**
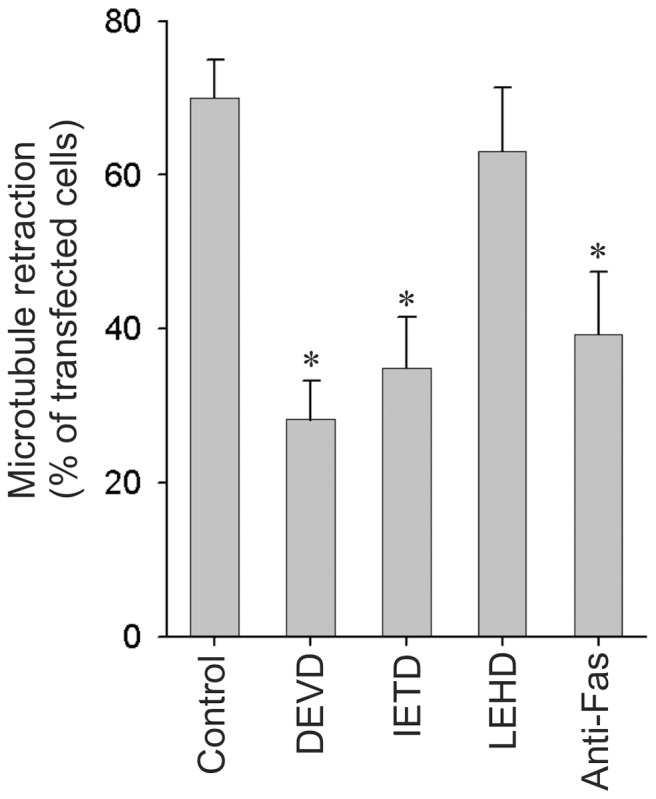
α-synuclein dependent degeneration in OLN-93 cells requires FAS signaling and caspase-8 activation. OLN-t40-AS cells stably expressing human α-syn were treated with peptide aldehyde inhibitors (20 µM) against caspase-3 (Ac-DEVD-CHO), caspase-8 (Ac-IETD-CHO), caspase-9 (Ac-LEHD-CHO) and FAS-blocking antibody (ZB4) (1 µg/ml) 1 h prior to transfection with p25α. MT retraction was quantified my immunofluorescence microscopy 24 h after transfection. Bars represent the mean ± standard error of mean (SEM) from three independent experiments. Inhibition of caspase-3, caspase-8 and FAS but not caspase-9 caused a significant reduction in MT retraction as compared with the control cells (*p*<0.05 with respect to untreated cells).

The involvement of caspase-8 and -9 in the activation of caspase-3 in this model was investigated by treating the cells with 20 µM of the peptide aldehyde inhibitors DEVD, IETD and LEHD, which are specific for caspase-3, -8, and -9, respectively. DEVD and IETD protected the cells from degeneration as demonstrated by a significant reduction in the number of transfected cells with MT retraction in contrast to LEHD that did not protect the cells (Fig. 1). This indicates that caspase-8 and caspase-3 are involved in p25α-induced degeneration in OLN-t40-AS cells. To corroborate that the caspase-8 dependency was due to an activation of FAS, we treated the cells with anti-FAS antibody ZB4, which acts as an antagonist to FASL binding to FAS. Pretreating the cells with ZB4 reduced the degeneration by approximately 45% (Fig. 1). This effect was dose–dependent, but could not be further enhanced (data not shown). These data demonstrate that activation of the death domain receptor FAS and downstream activation of caspase-8 and caspase-3 participate in the p25α-stimulated α-syn-dependent degeneration in oligodendrocytes.

### α-synuclein expression sensitizes primary oligodendrocytes to FAS ligand-dependent cytotoxicity

**Figure 2:**
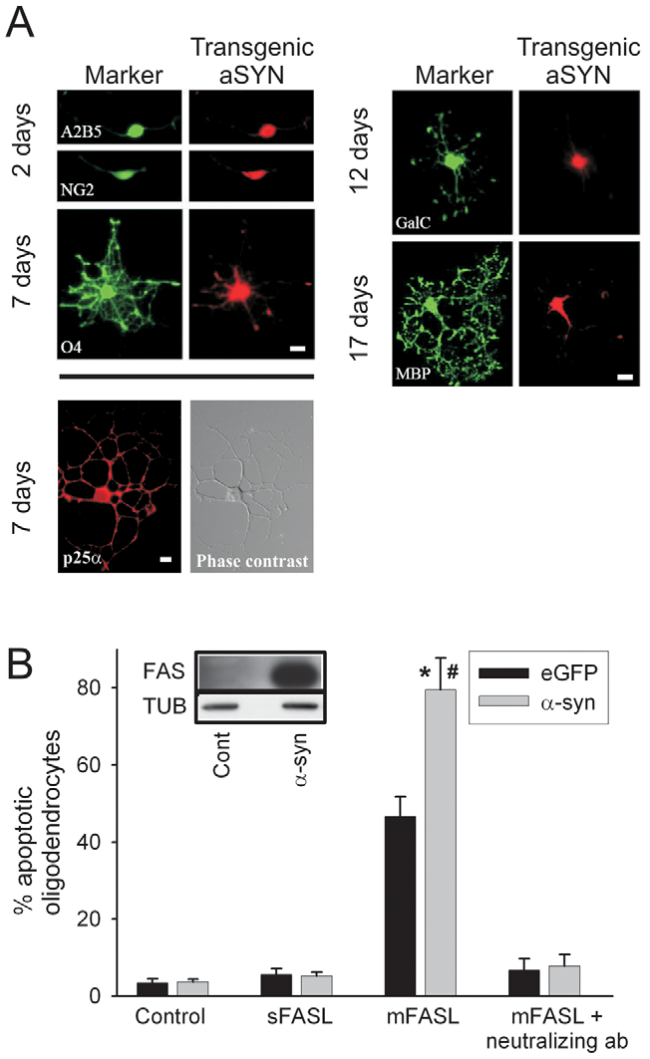
α-synuclein expressing oligodendrocytes are sensitized to FAS-dependent toxicity. **A,** *In vitro* differentiation of (PLP)-α-syn tg oligodendrocytes. Oligodendrocyte progenitor cells were isolated from tg mouse forebrains and cultured for the indicated times. Cells were fixed for double-label immunostaining with antibodies against A2B5, NG2, O4, GalC and MBP (green; left panels) as well as monoclonal 15G7 against human α-syn (red; right panels). Differentiated cells (7 DIV) were immunostained with an antibody against p25α and visualized by phase contrast microscopy. Scale bars, 10 µm. **B,** Human α-syn and eGFP tg oligodendrocytes were treated for 24 h with DMSO (control), sFASL, mFASL or preincubated for 30 min with FAS-blocking antibody before challenge with mFASL. Cells were fixed and labeled with O4 and Hoechst. mFASL treatment induced oligodendrocyte cell death as determined by apoptotic nuclei with α-syn oligodendrocytes being significantly more sensitive to FAS mediated cell death than eGFP oligodendrocytes. Data represent mean ± SEM of total tg oligodendrocytes from three independent experiments. Student *t* test (*n* = 3): *p<0.0001 compared with untreated cultures; #*p*<0.001 compared with eGFP tg cultures exposed to the same challenge. Western blots prepared from lysates of wild-type and α-syn tg oligodendrocyte cultures were sequentially probed with monoclonal anti-FAS and anti-α-tubulin antibodies (insert).

p25α is normally expressed in oligodendrocytes from the stage of myelination as demonstrated *in vivo* and during *in vitro* differentiation [23], [24], [25], [26]. By contrast, oligodendroglial α-syn expression occurs only in MSA and the presence of GCIs hallmarks this disease [27]. The oligodendroglial demise in MSA occurs gradually and discrete stages of dystrophic oligodendrocytes can be distinguished where expression of non-aggregated α-syn precedes the late aggregate-containing stage [9]. To investigate if FAS activation can be an active player in the pathophysiology of MSA, we derived enriched oligodendroglial cultures [20] from newborn (PLP)- α-syn mice [28]. These mice develop normally and display normal viability, although α-syn-expressing oligodendrocytes are sensitized to oxidative stress [7]. α-Syn was expressed and diffusely distributed throughout the cytoplasm in these cultures at all stages from the A2B5-positive or NG2-positive bipolar oligodendrocyte precursors, the O4 positive precursors to the MBP-positive, mature oligodendrocytes (Fig. 2A). The specificity of the 15G7 anti-α-syn antibody [29] was demonstrated by the absence of immunostaining in primary oligodendrocytes derived from (PLP)-eGFP tg mice (data not shown). Expression of endogenous p25α in these oligodendrocytes was demonstrated in oligodendrocyte precursors at 7 DIV (Fig. 2A), but it was also expressed in the later *in vitro* developmental stages (data not shown) in agreement with [24].

Fig. 2B demonstrates a low level of apoptosis in control cells from (PLP)-α-syn mice and the control strain (PLP)-eGFP as determined by Hoechst staining, which identified apoptotic cells. Challenging these primary oligodendrocytes with the ineffective, soluble form of FASL (sFASL) [30] did not stimulate apoptosis under these conditions (Fig. 2B). In contrast, an active preparation of FASL, provided as membranous vesicles (mFASL) [31], stimulated apoptosis in 47% of control eGFP-expressing oligodendrocytes and 80% of α-syn tg oligodendrocytes (Fig. 2B). Both were significantly increased compared with the untreated control cells, but apoptosis induction in the (PLP)-α-syn cells was significantly higher than in the control (PLP)-eGFP cells (*n* = 3; *p*<0.001). The apoptotic potency of mFASL was completely reversed using a FAS-blocking antibody (Fig. 2B). This corroborates the specificity of α-syn sensitization to FAS-mediated apoptosis in cultured oligodendrocytes.

The simplest mechanism by which oligodendrocytes would be sensitized to the apoptotic effects of FASL is up-regulation of the FAS receptor. Indeed, cell lysates from α-syn tg oligodendrocytes showed stronger FAS signal on immunoblot than cell lysates from control cultures (Fig. 2B, insert).

### Expression of FAS is increased in human MSA brain tissue

**Figure 3:**
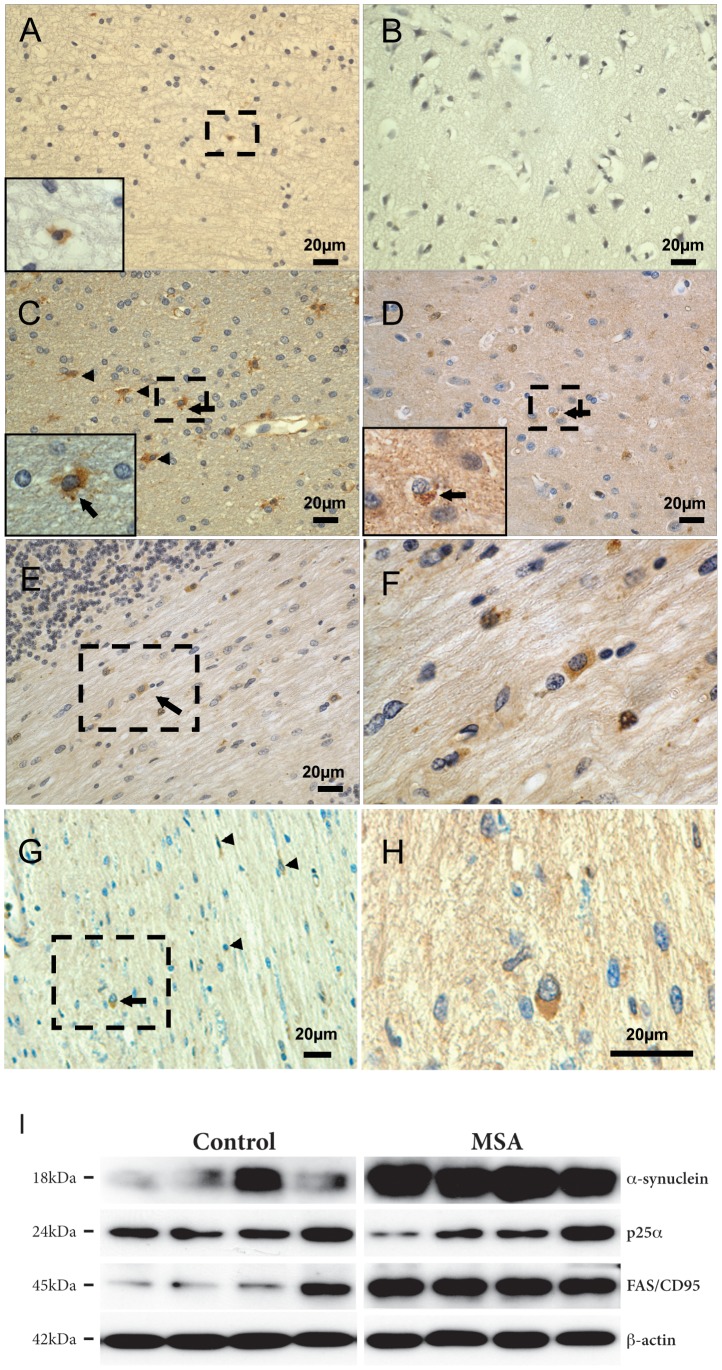
FAS is upregulated in human MSA brain. **A,** **B,** Immunohistochemical staining for FAS in normal controls. In normal controls there were only occasional FAS-positive cells shown here in (A) the inferior temporal cortical white matter and (B) grey matter. Insert in (A) shows a FAS-positive cell resembling a microglia. **C–H,** Immunohistochemical staining for FAS in MSA cases. MSA tissue contained numerous FAS-positive glial cells shown here in the inferior temporal cortical (C) white matter and (D) grey matter. Arrows indicate FAS-positive oligodendrocytes, and arrowheads indicate FAS-positive astrocytes or microglia. Inserts in (C) and (D) shows enlarged view of boxed regions showing FAS-positive oligodendrocytes (judged by their nuclear morphology). The oligodendrocyte in (D) contains an inclusion. E, FAS-positive glial cells in the cerebellar white matter. Boxed region in (E) is enlarged in (F) showing oligodendrocytes. G, FAS-positive cells in the pons. Arrow indicates a FAS-positive glial cell resembling a GCI-bearing oligodendrocyte. Arrowheads indicate FAS-positive astrocytes and microglia. Boxed region in (G) is enlarged in (H). **I,** Brain tissue (precentral gyrus white matter) from human MSA (*n* = 8) and control cases (*n* = 10) was sequentially extracted for TBS and SDS soluble proteins and the SDS soluble fraction were used for further analysis. Four MSA and four control cases are shown. Proteins (20 μg) were resolved by SDS-PAGE and analyzed by immunoblotting using antibodies against α-syn, p25α and FAS. Actin was included as a loading control. The molecular sizes (kDa) of the presented bands are indicated to the left. There was a significant increase in SDS-soluble FAS protein in MSA cases compared to controls (*p* = 0.043).

To validate the possible involvement of oligodendroglial FAS expression in MSA, we performed immunostaining on brain sections from neocortical, pontine and cerebellar regions from human brains. Normal control samples showed only occasional FAS immunoreactivity in glial cells in white and grey matter (Fig. 3A and B). By contrast, MSA cases showed numerous FAS-positive glial cells in both white and grey matter of the neocortical regions examined (frontal and temporal cortex), pons and cerebellum (Fig. 3C–H). In white matter regions, as exemplified by temporal cortex, pons and cerebellum, a robust labeling of a subset of GCI-bearing oligodendrocytes was evident along with numerous FAS-positive microglia and astrocytes (Fig. 3D and G–H). The immunostaining was specific as omission of primary antibody completely abrogated the staining (data not shown).

Increased expression of FAS was confirmed by immunoblotting of SDS-soluble extracts of brain homogenates from MSA cases and control subjects. All MSA cases had typical clinical disease and an average disease duration from diagnosis [32]. All had pathological α-syn-positive GCIs and an increase in SDS-soluble α-syn (Fig. 3I) consistent with previous literature [33]. A 45kD band representing FAS was observed and quantified. There was a significant increase (*p* = 0.043) in SDS-soluble FAS protein in MSA cases compared to controls (Fig. 3I). FAS levels among MSA cases did not correlate with α-syn tissue levels.

### Assessment of FAS, p25α and α-synuclein in GCIs in MSA

**Figure 4:**
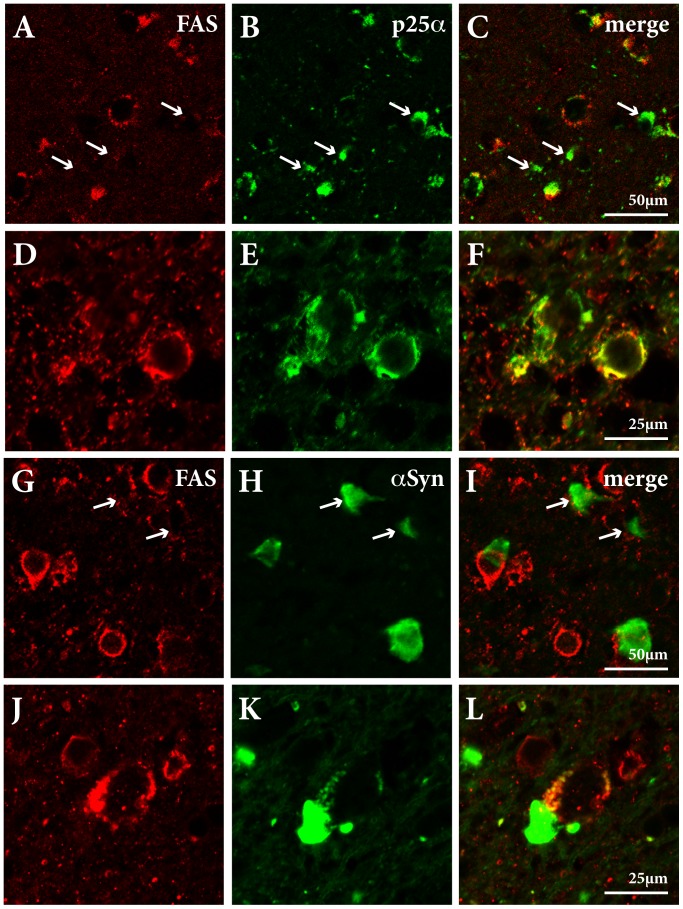
FAS colocalizes with p25α and α-synuclein in human MSA brain. Double labeling immunofluorescence using FAS (red) and p25α (green) antibodies (A–F) and FAS (red) and α-syn (green) antibodies (G–L) in putamen of an MSA case. Protein colocalization is shown as yellow in merged images (C, F, I, L). A–C, Identification of p25α-positive/FAS-negative GCIs (arrows) in oligodendroglia. D–F, Colocalization of FAS and p25α within GCI-like structures but not in small punctate cytoplasmic granules. G–L, FAS and α-syn colocalized in 4% of the GCIs (J–L) but the majority of α-syn-positive GCIs did not colocalize FAS (G–I, arrows indicate α-syn-positive/FAS-negative inclusions).

We have previously identified inclusions positive for p25α but negative for α-syn in approximately 50% of oligodendrocytes in MSA and α-syn-positive inclusions in the remaining oligodendrocytes [9]. The latter α-syn-positive cells (50%) are heterogeneous with around 60% coexpressing α-syn and p25α and the remainder expressing only α-syn. In the present study, we identified GCIs as either p25α-positive (Fig. 4A–F, arrows indicate p25α-positive/FAS-negative cells) or α-syn-positive in oligodendroglia (Fig. 4G–L). The p25α positive GCIs displayed two types of morphology, i) bright thin accumulations close to the nuclear membrane (Fig. 4E) or ii) more massive accumulations extending into the cytoplasm (Fig. 4B). By contrast, α-syn-positive inclusions displayed a morphology that resembles the massive p25α-positive inclusions (Fig. 4H, K). FAS was predominantly present as intense perinuclear labeling that extended into the cytoplasm as well as small punctate cytoplasmic granules (Fig. 4A, D, G, J). Double labeling immunofluorescence showed FAS and p25α immunoreactivity overlapping predominantly within perinuclear p25α-positive GCIs and to some extend in the more extensive cytoplasmic accumulations. However, p25α was not present in small FAS-immunopositive cytoplasmic granules in pathological oligodendrocytes, which resembles FAS staining of oligodendrocytes in MS lesions [34]. Double labeling immunofluorescence demonstrated that FAS and α-syn rarely colocalized within GCIs (Fig. 4I, L) but FAS was present in some oligodendrocytes harboring α-syn positive GCIs. Quantification of p25α-positive GCIs revealed that approximately 50% contained FAS immunoreactivity (Table 1). In contrast, the majority of oligodendrocytes with α-syn-positive GCIs (85%) did not contain FAS immunoreactivity (Fig. 4G–I, arrows indicate α-syn-positive/FAS-negative cells). Among oligodendroglia with α-syn-immunopositive GCIs, 11% contain FAS immunoreactivity outside the inclusion (Fig. 4G–I) while α-syn and FAS colocalized in only 4% of the GCIs (Fig. 4J–L and Table 1). Conclusively, FAS is frequently expressed in p25α-positive GCIs but only rarely in α-syn-positive GCIs. However, in the group of α-syn-immunopositive oligodendrocytes, FAS is mainly expressed outside the GCIs.

**Table 1:** Expression of FAS within p25α- or α-synuclein-positive oligodendrocytes in MSA.

Oligodendrocytes containing:	FAS+	FAS−
**p25α+ inclusions**	**210 (52%)**	**194 (48%)**
*FAS within inclusions*	*210 (100%)*	
**α-syn+ inclusions**	**58 (15%)**	**316 (85%)**
*FAS within inclusions*	*15 (4%)*	

*Human MSA brain tissue was analyzed for the presence of either FAS and p25α or FAS and α-syn using double immunofluorescense microscopy. A total of 404 p25α positive GCIs were identified and the presence of FAS colocalizing with p25α within the GCIs or FAS localized outside in the cytoplasm was determined by visual inspection. Similarly, 374 α-syn-positive GCIs were identified and scored as described above for colocalization with FAS or for the presence of FAS outside the GCIs in the cytoplasm*.

## Discussion

The mechanisms and signaling pathways involved in the degeneration of oligodendrocytes and neurons in MSA are still unclear although the aberrant accumulation of aggregated α-syn in oligodendroglial GCIs points to a central role of this molecule. This hypothesis has been corroborated by tg mouse studies where oligodendroglial expression of human α-syn causes overt neurodegeneration or sensitizes oligodendrocytes to the toxin 3-nitropropionic acid [4], [5], [6]. p25α is a protein that is expressed during oligodendrocyte differentiation from the state of myelination [23] and it stimulates α-syn aggregation *in vitro* [17]. Rat OLN-93 oligodendroglial cells express neither endogenous α-syn nor p25α but coexpression of the two proteins in these cells allowed cellular modeling of MSA-like degeneration that required both aggregation and phosphorylation at Ser129 of α-syn [19]. Using peptide inhibitors of caspase-3, -8 and -9, we demonstrate that caspase-8 and -3 inhibition potently protected the cells. FAS (CD95) is a death domain receptor that is activated by FASL, a transmembrane protein or as a soluble ligand depending upon proteolytic cleavage. FAS is capable of indirectly activating procaspase-8 and in the current study blocking FAS activation by the ZB4 antibody also protected the oligodendrocytes, demonstrating that α-syn aggregation initiates a cell autonomous autocrine signaling loop in the cell model.

Our hypothesis that coexpression of α-syn and p25α sensitizes oligodendrocytes to FAS-dependent apoptosis was investigated using primary oligodendrocytes cultured from oligodendrocyte precursors from tg mice expressing α-syn [4] or the control protein eGFP under the oligodendrocyte-specific PLP promoter. PLP-α-syn mice demonstrate a series of MSA-like abnormalities and show increased sensitivity to chemical stress, but no overt neurodegeneration [4], [7], [35]. The basal level of apoptosis was low in both α-syn and eGFP tg oligodendrocytes. However, treatment with microsomal preparations containing FASL induced a 40% increase in apoptosis in α-syn-expressing oligodendrocytes as compared to eGFP–expressing control cells. Apoptosis could be almost completely blocked by supplementing the cultures with FAS-blocking ZB4 antibody as observed for the OLN-93 model. The protective effect of blocking FAS signaling in the cellular MSA models resembles the *in vivo* protective effect of soluble FAS on oligodendrocytes after acute spinal cord injury in mice [36] and rat models [37]. Hence, *in vivo* trials will be justified attempting to block oligodendroglial FAS signaling in tg MSA mouse models either using FAS blocking therapeutics e.g. antibodies and soluble FAS, or crossing the MSA mice models with mice deficient in components of the FAS signaling system [16].

Post mortem MSA brain tissue is hallmarked by α-syn-containing GCIs and also characterized by neuron loss, myelin pallor and gliosis and thus represents the result of a complex degenerative process. We demonstrate for the first time that FAS expression is increased in neocortical, cerebellar and pontine MSA tissue from both grey and white matter with strong staining in oligodendrocytes and also in astrocyte- and microglia-like cells. We previously proposed a staging of GCI development in the course of MSA-associated oligodendroglial degeneration [9]. This staging is based on dividing GCIs into four stages representing the progressive accumulation of 1) p25α (approximately 50%), followed by 2) α-syn (approximately 25%), which subsequently is transformed into 3) amyloid-type aggregates (approximately 7%). Finally, the cells shrinks 4) and lose p25α expression (13%). Here we demonstrate that FAS is present in 50% of oligodendrocytes harboring p25α-positive inclusions where FAS and p25α colocalize within the inclusions. The localization of FAS within the thin perinuclear p25α-positive GCIs, likely representing type 1 inclusions, indicates that FAS is expressed early in the degenerative process of the oligodendrocyte. This is corroborated by low FAS expression in oligodendrocytes with α-syn-positive GCIs (types 2–4).

Human MSA brain tissue retains its number of oligodendrocytes despite the pronounced nerve cell loss and astrogliosis, which is suggestive of an enhanced turnover of oligodendrocytes from oligodendroglial precursor cells [38]. The predominant expression of FAS on oligodendrocytes exhibiting early stage type 1 GCIs may indicate that FAS activation could be an active player priming the oligodendrocytes for degeneration as observed in active MS lesions with enhanced FAS expression [34]. Early FAS stimulation can hypothetically contribute to the overall demise of the cell but also to the enigmatic expression of α-syn in oligodendrocytes, either by enhanced cellular uptake from the extracellular fluid or by activating α-syn gene expression. This may be similar to the enhanced α-syn expression in substantia nigra neurons adjacent to degenerating neurons in a striatal lesion model. This model has provided evidence that soluble mediators from degenerating cells can stimulate α-syn expression [39].

The mechanism behind the neuronal loss in MSA is not clear but the enhanced expression of FAS early in GCI formation along with the expression in astrocytes and microglia, suggests that FAS mediated signaling may contribute to a broader prodegenerative tissue remodeling e.g. via inflammatory NF-κB activation [40].

In conclusion, MSA-associated neurodegeneration is associated with FAS expression in oligodendrocytes displaying early GCI lesions along with astrocytes and microglia. Mechanistically, α-syn tg cell line and oligodendrocyte models of MSA display an enhanced sensitivity to FAS dependent signaling. This suggests that manipulation of this signaling system may hold promise as a therapeutic target in this yet intractable disease.
